# Study protocol for a randomized controlled trial evaluating the effectiveness of an internet-based self-help intervention to cope with psychological distress due to COVID-19 in the Italian general population: the RinasciMENTE project

**DOI:** 10.1186/s13063-022-06714-x

**Published:** 2022-09-24

**Authors:** Vanessa Bertuzzi, Michelle Semonella, Gerhard Andersson, Gian Mauro Manzoni, Gianluca Castelnuovo, Enrico Molinari, Giada Pietrabissa

**Affiliations:** 1 Department of Psychology, Catholic University of Milan, 20123 Milan, Italy; 2 Department of Psychology, Bar-Ilan University, 52900 Ramat-Gan, Israel; 3 Department of Behavioural Science and Learning, Linköping, Sweden; 4 Department of Clinical Neuroscience, Karolinska Institute, Solna, Sweden; 5 Department of Psychology, Faculty of Psychology, eCampus University, 22100 Como, Italy; 6 Psychology Research Laboratory, Istituto Auxologico Italiano IRCCS, Milan, Italy; 7 Università Cattolica del DSacro Cuore, Via Largo Gemelli, 1, 20123 Milan, Italy

**Keywords:** COVID-19, Internet-based intervention, Self-help, Cognitive-behavioral therapy, Psychological distress, Randomized controlled trial, Clinical psychology

## Abstract

**Background:**

This study aims to evaluate the feasibility and effectiveness of the RinasciMENTE program, an Internet-based self-help intervention based on cognitive behavioral therapy (CBT) principles and techniques in supporting individuals experiencing psychological impairments during the COVID-19 pandemic. A randomized controlled trial (RCT) design with random allocation at the level of individual will be conducted to compare the impact of the RinasciMENTE program with a waiting list control in improving the psychological functioning of the general population during the COVID-19 pandemic.

**Methods:**

A minimum sample of 128 participants experiencing mild/subthreshold levels of psychological symptoms during the COVID-19 pandemic will be recruited. After the initial screening, participants will be randomly assigned to either the experimental group or the control condition. The program will last 2 months, during which participants will receive 8 weekly CBT treatment modules. The impact of the RinasciMENTE program on selected primary and secondary psychological outcomes will be tested at the end of the intervention (2 months) and 6- and 12-month follow-ups.

**Discussion:**

We expect people to show an increased level of psychological functioning and to acquire the skills and self-confidence necessary to deal with the psychological consequences of the COVID-19 outbreak and its related social isolation during and following the pandemic.

**Trial registration:**

ClinicalTrials.gov NCT0497903 Registered on 28 May 2021

## Background

In the hope of reducing the transmission of coronavirus disease 2019 (COVID-19) governments around the world have implemented unprecedentedly strict preventive measures, such as home confinement, mobility restrictions, and social distancing [1, 2]. However, the COVID-19 pandemic and the resulting economic recession [3] have negatively affected the mental health of many people [4]. The most common mental disorders emerging were anxiety and depression, obsessive-compulsive symptoms, insomnia, and post-traumatic stress disorder symptoms [5–9]. These were not only a direct consequence of the pandemic but also largely driven by the effects of prolonged social isolation. Although necessary to limit the spread of the epidemic, social isolation resulted in increased sedentary lifestyle [2] and other COVID-19-related stressors including fears of infection, frustration and boredom, and lack of in-person contacts [10, 11]. Italy was one of the most affected countries during the outbreak, initially accounting for over 223,000 infected individuals and more than 31,000 deaths [12]. In May 2021, 4,111.,110 people resulted having contracted the virus, and 122,833 of them died as a consequence [13, 14].

In this scenario, it is crucial to assess the level of psychological distress and to provide ad hoc psychological interventions to support the individuals’ wellbeing in both the general population [15, 16] and at-risk groups [17, 18] during the COVID-19 pandemic.

Accordingly, evidence from previous epidemics highlights the risk for long-term mental health issues [19] and emphasizes the need for continued support during and after the pandemic [20].

Many health organizations have already committed resources to support the mental well-being of the individuals, adapting existing standard programs to meet evolving demands caused by COVID-19 [21]. In this regard, cognitive-behavioral techniques including restructuring of cognitive bias, as well as activity planning and relaxation, have been shown particularly useful in addressing psychological distress [22–25].

Cognitive-behavioral therapy (CBT) is a time-sensitive, structured, present-oriented psychotherapy aimed at helping people identify and change thinking and behavior patterns that are harmful or ineffective, replacing them with more accurate thoughts and functional behaviors.

Still, given that face-to-face treatment is not always viable in these circumstances [26], a new way to deliver psychological treatments is urgently required, and a potentially practical solution is to deliver therapy remotely [14, 27–30].

Many systematic reviews and randomized controlled trials (RCTs) demonstrated the utility and the efficacy of Internet-based interventions in supporting people experiencing psychological problems [31–33].

Advantages include improved access for individuals [26] who have variable schedules, an important workload, or who are afraid of being contaminated through face-to-face contacts [34–36], as well as cost-effectiveness compared to face-to-face treatment.

Due to its features, CBT well-suits many aspects of distance therapy. First, it is a talking therapy, and this aspect can be relatively easily retained remotely. It also emphasizes the importance of the person making changes and working on specific tasks between sessions to bring about the change [37]; this is perfectly consistent with working remotely. Moreover, with distance therapy, the patient may be less likely to attribute the progress to the therapist and more likely to have an improved sense of self-efficacy [38]. One of the goals of CBT is to “become your therapist” by learning skills people can use on their own after treatment to keep feeling well.

Research shows that CBT delivered digitally (iCBT) is effective in reducing symptoms of social anxiety disorder [39, 40], generalized anxiety disorder [41, 42], panic disorder [43], major depressive disorder [44–46], obsessive-compulsive disorder [47], and insomnia [48] in both guided and unguided self-help programs. Specifically, a review of 30 studies found that CBT-based self-help interventions significantly reduced both anxiety and depression. Studies also show that people tend to maintain their progress over time, which is very encouraging [49]. Furthermore, since patients can return to the program at their convenience to access treatment information, this may facilitate learning and retention.

Despite a growing interest in the field of web-based psychological interventions, Internet-based CBT self-help programs remain underdeveloped.

To fill this important gap in the literature and to properly meet the need of the population, it is crucial to promote and investigate the impact of this Internet-based psychological intervention in increasing individuals’ emotional well-being.

For this reason, the present RCT aims to explore the effectiveness of a novel Internet-based CBT self-help program specifically developed to address the immediate stress and prevent long-term psychological consequences of the COVID-19 pandemic among Italians both living in the county and abroad. To assess its impact, the effects of the RinasciMENTE program will be compared with a waiting list (WL) condition on selected self-reported measures immediately following the 8-week intervention and at 6- and 12-month follow-ups.

The primary hypothesis that will be tested is that the program will be feasible and effective in increasing the psychological functioning of the person.

The secondary hypothesis will test whether participants assigned to the experimental condition will show a reduced level of stress, anxiety, depression, and fear of COVID-19, as well as improved emotion regulation strategies, self-efficacy, and psychological well-being than those in the WL condition at treatment termination.

Moreover, participants in the experimental condition will be expected to present maintained outcomes or further decreased psychological symptoms at 6 and 12-month after the end of the treatment program.

## Methods

### Design

The project consists of a prospective, randomized, open, and parallel group-controlled study with two arms: an experimental arm with 8 online weekly CBT self-help sessions, and a waiting list (WL) control arm. Ours is a superiority trial as it is aimed at testing the superiority of the online intervention compared to the waiting list.

### Ethical statement

The study was approved by the Ethical Committee of the Catholic University of Milan, Italy (ID: 25-21). All procedures performed in the study will be run following the ethical standards of the institutional and/or national research committee and with the Helsinki Declaration and its later amendments or comparable ethical standards.

### Patients and public involvement

Participants from the general population will be recruited through advertisements placed on social media platforms (i.e., Facebook, Instagram, Twitter) and online webinars on the topic.

The recruitment materials will include details about the study aim, the treatment delivered, and the conditions for participation, together with the weblink to access the program.

Inclusion criteria for the participants into the study will be as follows: (A) being fluent in the Italian language from different countries; (B) being over 18 years old; (C) providing online informed consent; and (D) showing mild/subthreshold levels of symptoms at the 12-Item General Health Questionnaire (GHQ-12)—Italian version [50, 51]—the most extensively used screening tool assessing the severity of common mental disorders over the past few weeks using a 4-point Likert-type scale (from 0 to 3). Its total score ranges from 0 to 36, with high scores indicating worse health status. Only participants who score ≤ 19 on the GHQ and show moderate levels of symptoms at the clinical interview will be included in the study.

Participants will be excluded from the program if (A) presenting visual, hearing, or cognitive impairments that will prevent them from receiving and following the intervention; (B) suffering from severe psychiatric disorders according to the Diagnostic and Statistical Manual of Mental Disorders (DSM 5) [52]; and (C) lacking basic computer skills or internet access.

Respondents will not be excluded if already receiving psychopharmacological therapy or psychological-psychotherapeutic support.

### Sample size calculation

The minimum sample size required to conduct this study was calculated using an a priori sample size calculator (G*Power 3.1.9.2 software) for *F* tests [53–55]. Participants were randomly divided into two groups: (A) iCBT self-help and (B) waiting list control. Moreover, participants will be measured in 3 moments: (1) before the intervention, (2) at the end of intervention (2 months later), and (3) after 12 months from treatment termination. Due to the novelty of the study from which to derive realistic estimates of effect sizes, the partial *η*^2^ was set a priori to assume a value of 0.02—small effect size [56, 57]—which provides a Cohen’s *f* value of 0.143. Moreover, the type I error (*α*) rate was set at 0.05 (two-sided), and the power (1 – *β*) was set at 0.95, and the a priori correlation between repeated measures was set at 0.50, according to general guidelines [56]. Lastly, the non-sphericity correction was set to 1. The results showed that there is a 95% chance of correctly rejecting the null hypothesis of no significant effect of the interaction with 128 participants in total (64 for each group).

### Randomization and blinding

**Fig. 1:**
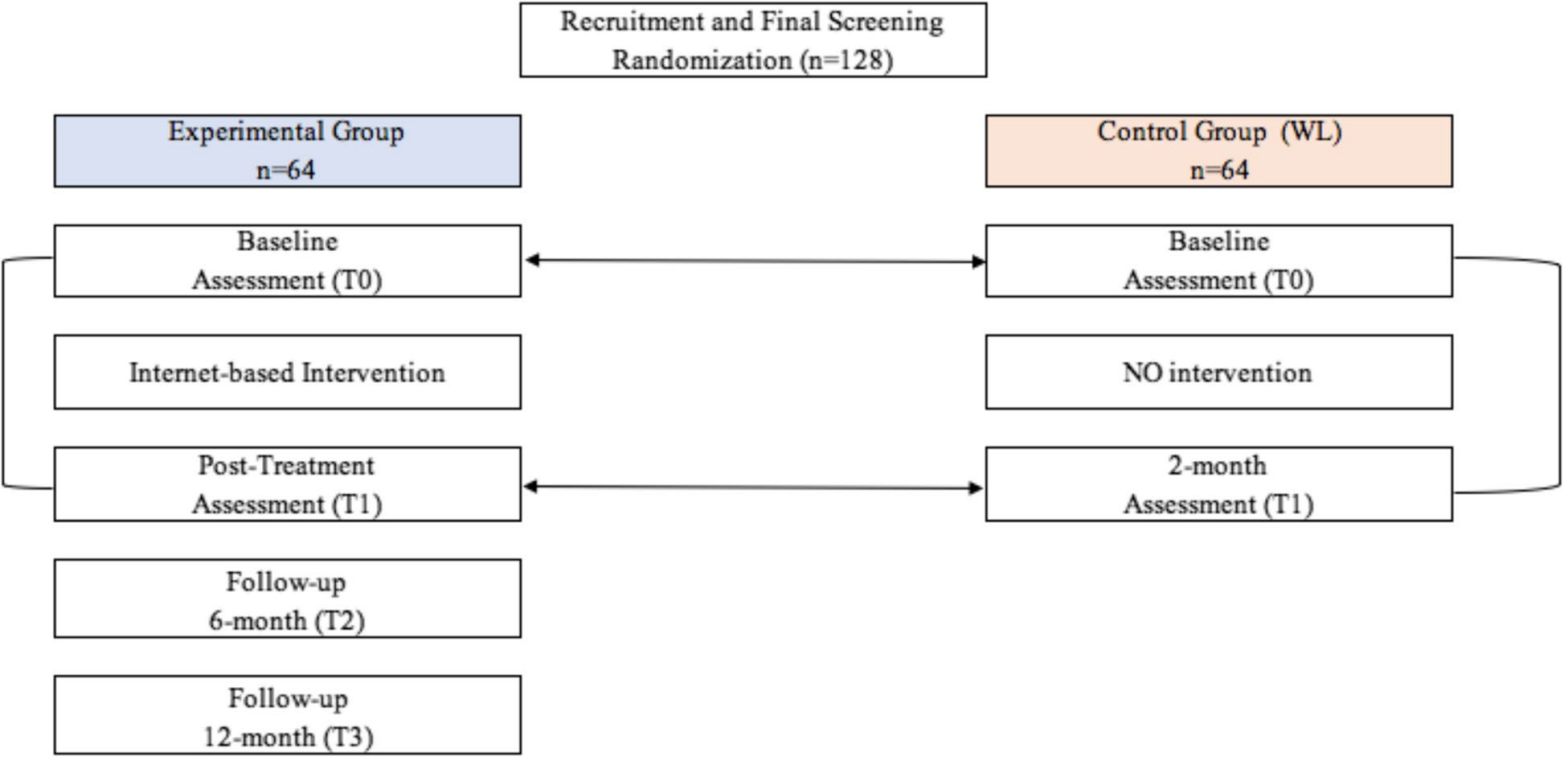
Flow chart of the RinasciMENTE study

The random randomization scheme and the allocation will be generated using the website Randomization.com [58], and the responsible will be one of the two researchers involved in the program. Allocation concealment will be ensured by the program generating an anonymous code for each participant that will be associated with the randomization sequence. Due to the nature of the intervention, the treatment group allocation cannot be concealed from the participants nor the research team and assessor of outcomes. The clinical psychologist who will conduct the sessions, the participants, and the observers will be blind to the research aims. Participants will be assigned to one of two conditions within 2 working days from their baseline assessment (Fig. 1).

### Measures

#### Demographic and clinical information

Information about age, gender, education, civil status, weight, and high—used to calculate the body mass index (BMI, kg/m^2^) in presence of dysfunctional eating behaviors—will be self-reported online at baseline. Subjects will be also asked to provide information about the country where they are currently living, their work situation (before and after the COVID-19 pandemic), and the personal experience they made of both COVID-19 pandemic and related preventive measures.

**Fig. 2:**
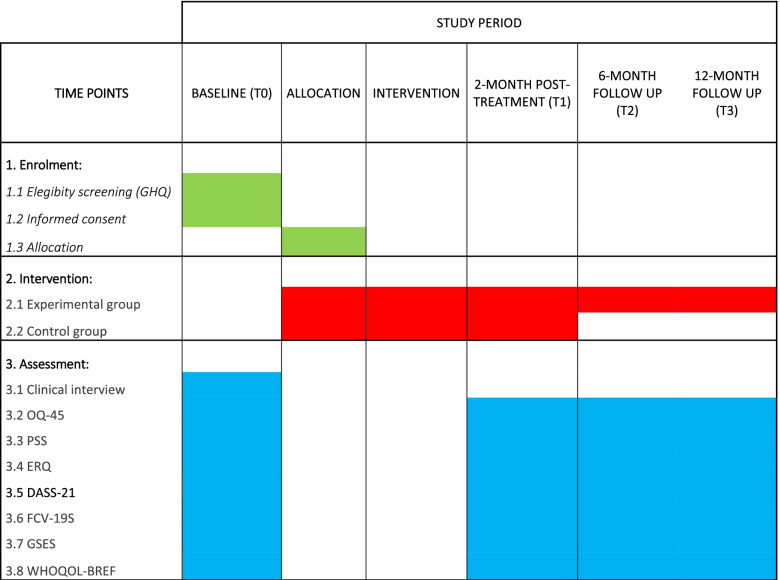
Gantt of the RinasciMENTE study

The Italian version of the following psychological measures will be also collected at baseline (T0), treatment termination after 2 months (T1), and at 6-month (T2) and 12-month (T3) follow-ups in the experimental condition only. Please see Fig. 2 for a detailed project timeline.

#### Primary outcome

*Outcome Questionnaire (OQ-45)* [59] composed by 45 items rated on a 5-point Likert scale (0 = never – 4 = almost always) assessing the treatment progresses across 3 different domains: symptom distress (SD—25 items: 2, 3, 5, 6, 8, 9, 10, 11, 13, 15, 22, 23, 24, 25, 27, 29, 31, 33, 34, 35, 36, 40, 41, 42 and 45), interpersonal relations (IR—11 items: 1, 7, 16, 17, 18, 19, 20, 26, 30, 37, and 43), and social role (SR—9 items: 4, 12, 14, 21, 28, 32, 38, 39, and 44). The OQ-45 total score is calculated by summing the score of the three subscales. The total score (range 0 to 180) is calculated by the sum of the items: a total score ≥ 63 identifies the presence of clinically significant symptoms. Specifically for the subscales, a score ≥ 36 in the first subscale indicates significant symptoms of distress, a score ≥ 15 in the second subscale indicates dysfunctional relationships, and a score ≥ 12 in the third subscale indicates the presence of an ill-defined social role.

We expect that the primary outcome will change from baseline: the results will be aggregated using mean and standard deviation.

#### Secondary outcomes

The *perceived stress scale* (PSS) [60] is composed of 10 items rated on a 5-point Likert scale (from 0 = never to 4 = very often) assessing the degree to which situations in one’s life are appraised as stressful. PSS scores are obtained by reversing responses (i.e., 0 = 4, 1 = 3, 2 = 2, 3 = 1, and 4 = 0) to the four positively stated items (items 4, 5, 7, and 8) and then summing across all scale items. The total score (range 0 to 40) is calculated by the sum of the items.

The *Emotional regulation questionnaire (*ERQ) [61] is composed of 10 items rated on a 7-point Likert scale (from 1 = strongly disagree to 7 = strongly agree) assessing respondents’ tendency to regulate their emotions in two ways: cognitive reappraisal (items 1, 3, 5, 7, 8, and 10) and expressive suppression (items 2, 4, 6, and 9). The total score (range 7 to 70) is calculated by the sum of the items.

The *Depression Anxiety Stress Scales-Short Version* (DASS-21) [62] is composed by 21 statements rated on a 4-point Likert scale (from 0 = did not apply to me at all to 3 = applied to me very much) assessing the negative emotional states of depression, anxiety, and stress. Each of the three DASS-21 scales contains 7 items: *Depression*: dysphoria, hopelessness, devaluation of life, self-deprecation, lack of interest/involvement, anhedonia, and inertia (items 3, 5, 10, 13, 16, 17, and 21); *Anxiety*: autonomic arousal, skeletal muscle effects, situational anxiety, and subjective experience of anxious affect (items 2, 4, 7, 9, 15, 19, and 20); and *Stress*: levels of chronic nonspecific arousal, difficulty relaxing, nervous arousal, and being easily upset/agitated, irritable/over-reactive and impatient (items 1, 6, 8, 11, 12, 14, and 18). Sum scores are computed by adding up the scores on the items per (sub)scale and multiplying them by 2. Sum scores for the total DASS-total scale thus range between 0 and 126, and those for each of the subscales may range between 0 and 42. Ranges of scores correspond to levels of symptoms, ranging from “normal” to “extremely serious.” Notably, the DASS-21 has been recently used to investigate the impact of the COVID-19 pandemic on the physical and mental health of different populations worldwide [63–65].

The *Fear of COVID-19 Scale* (FCV-19S) [66] is composed by 7-item rated on a 5-point scale, ranging from 1 (from 1 = strongly disagree to 5 = strongly agree) reflecting cognitive, emotional, behavioral, and physiological manifestations of fear related to COVID-19. The total score (range 7 to 35) is calculated by the sum of the items. All items are positively worded, implying that higher scores indicate greater levels of fear.

The *General Self-efficacy Scale* (GSES) [67] is composed of 10 items rated on a 4-point Likert scale (from 1 = not at all true to 4 = exactly true) assessing the individuals’ confidence in their ability to cope with a variety of difficult or stressing situations. The total score is calculated by finding the sum of all items. For the GSE, the total score ranges between 10 and 40, with a higher score indicating more self-efficacy.

The *World Health Organization Quality of Life (WHOQOL)-BREF* [68] is composed of 26 items rated on a 5-point Likert scale assessing four domains: physical health (7 items on mobility, daily activities, functional capacity, energy, pain, and sleep), psychological health (6 items on self-image, negative thoughts, positive attitudes, self-esteem, mentality, learning ability, memory concentration, religion, and the mental status), social relationships (3 items on personal relationships, social support, and sex life), and environmental health (8 items related to financial resources, safety, health, and social services); it also contains QOL and general health items. The scores (range 25 to 130) are transformed linearly to a 0–100-scale.

Moreover, *adherence* will be measured through indicators such as the percentage of visited pages or the number of accesses.

A*cceptability* will be assessed by registering the participants’ attrition rates and—for the experimental group only—by the use of the *System Usability Scale* (SUS) [69] composed of 10 statements scored on a 5-point Likert scale (from 1 = strongly disagree to 5 = strongly agree). The final score range from 0 to 100, with a score above 68 indicating the absence of usability problems.

### Procedure

Those interested in taking part in the program will be directed to the RinasciMENTE website (https://www.iterapi.se/sites/rinascimente/login) where they will find the written (online) consent, and after the acceptance of this, to each participant will be sent a document with information on the aims and requirements of the study.

They will be first asked to complete the GHQ-12 self-report questionnaires. Then, during the following 5 days, they will choose a time slot to be contacted for a clinical semi-structured interview lasting about 45 min, led by a certified clinical psychotherapist not involved in the study who will further assess the individual’s eligibility to take part in the study. Additional information about the study and the randomization procedure will be also given to all respondents.

Next, the inclusion of each participant in the study and modules comprising the program will be discussed in a group by the professional who will conduct the interview and the research investigators (GP and VB), and the decision will be e-mailed to each participant within a week. Reasons for exclusion will be explained to the persons and—in presence of severe psychological disturbance—individuals will be suggested to ask for professional help.

Eligible subjects will be then randomly assigned to either the experimental (iCBT self-help) or control group (WL) and will receive an e-mail with a username and a customized link to create their password allowing them to log into the *iterapi* platform [70, 71]. The *iterapi* is a safe platform with demonstrated efficacy in reducing symptoms of a wide range of disorders including somatic problems—such as hearing loss [72] and tinnitus [73]—to more traditional forms of psychological suffering, such as anxiety [74] and depression [75].

Participants in the experimental group will be required to make their first access to the platform and sign an electronic informed consent form within 24 h, while those in the control condition will be able to access the online treatment once the experimental group will conclude the intervention (after 2 months).

The platform will be used for communication between the mental health professionals and the participants, for the delivery of the intervention, and the quantitative assessments.

Before entering the program, participants in the study will have to complete the self-report questionnaires assessing the above selected primary and secondary outcomes (T0).

The treatment will consist of 8 weekly modules, for a total duration of 2 months. Seven modules (*introduction*, *behavioral activation*, *dysfunctional beliefs*, *acceptance*, *stress management*, *problem resolution*, and *plan of completion and maintenance*) will be maintained for all participants, while one module will be selected tailored on the specific psychological needs of each participant between the following: (1) *emotional school*, (2) *anxiety and exposition*, (3) *constant anxiety*, (4) *social anxiety*, (5) *panic attack*, (6) *sleep quality*, (7) *perfectionism*, (8) *relaxation*, and (9) *management of difficult memories*. The description of each module is reported in Table 1.

**Table 1:** Description of the RinasciMENTE modules

#	Title	Content
1	Introduction	In this module, the possible psychological consequences of the COVID-19 will be first discussed. Then, participants will be provided with information about the program. Also, a brief introduction about the principles and techniques of CBT will follow. Lastly, participants will be asked to start focusing on their life values and to start thinking about how these might influence their psychological wellbeing.
2	Behavioral activation	This module aims to increase the persons’ understanding of the connection between the individuals’ feelings and behaviors. Both enjoyable and unpleasant life activities will be also identified, to increase the frequency, duration, and quality of the pleasant ones.
3	Dysfunctional beliefs	This module explains how negative thoughts can influence mood and how to manage the cognitive traps using alternative thinking strategies.
4	Acceptance	In this module, the “disease paradox” will be introduced, followed by the cognitive-behavioral concept of acceptance—that is having more time for oneself. Then, the life values of the person will be identified and aligned to the therapeutic aim(s).
5	Emotional school	This module will provide the person with a comprehensive understanding of how emotions work and from where they originate. Then, participants will be taught how to manage negative emotions.
6	Anxiety and exposition	This module explains what anxiety is, how it manifests itself, and how to deal with it. Then, the person will be asked to create a list of situations triggering anxiety, and then to try to be progressively expose to these conditions.
7	Constant anxiety	This module explains what anxiety is, how it manifests itself, and how to deal with it. Then, what is meant for worries and how to exert control over them will be further discussed.
8	Social anxiety	This module explains what anxiety is, how it manifests itself, and how to deal with it. Then, the person will be asked to create a list of social situations triggering anxiety, and then to try to be progressively exposed to these conditions.
9	Panic attack	This module explains what anxiety is, how it manifests itself, and how to deal with it. Then, the person will learn to use hyperventilation and diaphragmatic breathing strategies when exposed to triggering stimuli.
10	Sleep quality	In this module, factors influencing the individuals’ sleep quality will be discussed, together with strategies aimed to modify dysfunctional habits for better sleep quality.
11	Perfectionism	This module explains what useless perfectionism is, and what are common beliefs and behavioral perfectionist models. Then, different strategies (cost of perfectionism and self-compassion) to manage useless perfectionism will be explained to the persons.
12	Stress management	This module explains what stress is and teaches the person two strategies (give themselves time to recover, and strategic event planning) to cope with stressful situations. Then, the person will be asked to choose and to use one of these two strategies.
13	Relaxation	This module explains what the “general tension” is and how to use diaphragmatic breathing. The module also describes how to reach relaxation through muscle control.
14	Problem resolution	This module aims to identify the problems in a person’s daily life. Different personalized solutions will be found for each problem, and the person will be asked to choose and to use one of them.
15	Management of difficult memories	This module describes what happens in the brain during stressful events and provides participants with two strategies (write drown difficult memories, and “stay in the memories” even if it seems overwhelming) to cope with difficult memories. Then, participants will be asked to choose and use one of these two strategies.
16	Plan of completion and maintenance	This concluding module explains how to maintain the achieved progress and cope with possible relapses.

For the entire duration of the intervention, every Thursday participants will receive an e-mail communicating the update of the new material on the platform. Participants who will not access the material or that will not complete the suggested tasks and exercises will receive a weekly reminder containing a brief encouraging message every Monday.

At the end of the program (after 2 months—T1), participants in both conditions will be asked to fill in the baseline questionnaires again. Then, the experimental group will be also assessed after 6 and 12 months from the term of the treatment (T2 and T3) (Fig. 1).

We do not expect any adverse or unintended effects due to the trial participation. However, in case of any form of psychological discomfort, participants can consult the psychologist responsible. Moreover, in case of any doubts or need for information, participants can contact the responsible of the study. Once enrolled, patients may withdraw from the study at any stage and this will not affect their future treatment.

### Statistical analysis

Statistical analyses were performed using SPSS software ver. 24.0 [76].

First, preliminary analyses will be performed to test the assumptions of both univariate and multivariate normality: if (strong) violations will be detected, robust methods or data transformation will be applied. Dropouts will be excluded from the study in this preliminary analysis.

A missing values analysis will be run to see if the values are missing completely at random (MCAR) or if there is some pattern among missing data. If there are no patterns detected, then pairwise or listwise deletion will be done to deal with missing data. However, if the analysis of the missing value detects a pattern, then imputation will be done.

The demographic characteristics will be reported as means and standard deviations for continuous variables and frequencies and percentages for categorical variables.

The chi-square statistic will be used to test the association between treatment groups and socio-demographic and variables (i.e., age, gender, education, civil status, job), and correlation analysis will be used to test the association between quantitative variables.

The outcomes will be compared in the two treatment conditions using an intention-to-treat (ITT) approach. The significance level will be set at 5%.

A repeated-measures ANOVA will be used to determine whether there are any between- and within-group differences in the selected outcomes from baseline to treatment termination. Then, repeated-measures within group ANOVA will test outcome differences over time baseline, end of treatment, and 6- and 12-month follow-ups in the experimental group only. Corrected effect sizes (Cohen’s *d*) and significance at 95% confidence interval (95% CI) will be calculated for both between-group and within-group differences, while for the total effect will be calculated the Cohen’s *f*.

Analysis will be performed by an independent statistician, blinded for the treatment allocation—who will discuss the results with the research investigators in a joint meeting.

## Discussion

To help and mitigate the impact of the COVID-19 pandemic on selected psychological parameters, the objective of this study is to evaluate the effectiveness of an Internet-based self-help intervention based on CBT principles and techniques, in reducing the level of stress, anxiety, depression, and fear of COVID-19, while increasing emotions self-regulation and perceived self-efficacy of Italians both living in the country and abroad.

The online modality was chosen due to the current multiple barriers for face-to-face psychological interventions (i.e., mobility), and its largely acknowledged advantages, including greater flexibility or the possibility to reach rural or low-income population [77], and Italians living outside the country who look for psychological support in their language. Also, the self-help approach to therapy will be employed to further improve access to treatments and empower users to strengthen their self-management ability, while putting less strain on therapeutic resources [78] than conventional treatments do.

### Expected results

The results of this RCT will provide evidence for the feasibility and effectiveness of the RinasciMENTE program in supporting the emotional well-being of the persons. Specifically, people are expected to show reduced levels of stress, anxiety, depression, and fear of COVID-19, as well as improved emotion regulation strategies, and self-efficacy. They are also expected to acquire the skills necessary to deal with the psychological consequences of social isolation during and after the COVID-19 outbreak. Furthermore, the levels of adherence and drop-out represent our criteria for defining the feasibility of the study.

Still, among the weaknesses of this study, it must be anticipated possible lack of internet access and digital literacy skills of some people.

Non-digital natives (i.e., older people) might, indeed, experience difficulties in the use of digital tools, and social isolation can pose additional challenges to their usage (i.e., chaotic home environments, limited privacy, or unreliable internet connection) [79].

The use of online intervention might also prevent effective controlling of confounding variables (i.e., environmental factors) that might impact treatment outcomes [80]. For this reason, before the start of the trial, a usability analysis involving a little sample of people from general population will be carried out in order to evaluate any aspects of difficulty in using the platform and thus be able to resolve them in time.

The results from this study will help to detect and address any usability problem, as Internet-based interventions, due to their characteristics and format, might be a suitable and feasible solution to mitigate the psychological impact of the virus outbreak and its related containment measures on the mental health status of the general population across the lifespan. Moreover, the findings will contribute to the further adaption of the self-help CBT programs.

In line with previous research findings, it is possible to predict that dropout rates may be higher with web-based interventions than in traditional face-to-face therapy [80], especially when self-help programs are used. To overcome this problem, participants in the study will receive a weekly reminder and motivational messages to access the online material. A positive relationship between each respondent and their referral therapist will be also supported, and while on the one hand, the professional remains available in case of need, the subject takes responsibility over the course of the treatment. The learning process is emphasized, so to help the person improve self-management skills and strategies to deal with their emotional problems.

Furthermore, the online format brings with it the advantage of allowing us to also support the population of Italians abroad, of which the literature does not report any in-depth analysis. RinasciMENTE, therefore, represents the first attempt to provide online psychological support to the segment of the Italian population abroad.

## Conclusion

Since the beginning of the COVID-19 pandemic, studies demonstrated a significant clinical and statistically decrease in mental health across populations worldwide. This burden is expected to continue in the aftermath making high demand for timely and pragmatic psychological interventions tailored to the unique and immediate needs of the general public. Preventive measures, fear of being infected, and financial losses give a reason for the implementation and use of digital self-help programs based on CBT principles and techniques—which might offer a range of self-management strategies to cope with emotional difficulties, besides reducing the cost of the health care system [81].

## Data Availability

All data will be identified only by a code, with personal details kept in a secure online platform with access only by the immediate research team.

## References

[1] Bonaccorsi G, Pierri F, Cinelli M, Flori A, Galeazzi A, Porcelli F (2020). Economic and social consequences of human mobility restrictions under COVID-19. Proc Natl Acad Sci U S A.

[2] Pietrabissa G, Volpi C, Bottacchi M, Bertuzzi V, Guerrini Usubini A, Loffler-Stastka H (2021). The impact of social isolation during the COVID-19 pandemic on physical and mental health: the lived experience of adolescents with obesity and their caregivers. Int J Environ Res Public Health.

[3] Xiong J, Lipsitz O, Nasri F, Lui LMW, Gill H, Phan L (2020). Impact of COVID-19 pandemic on mental health in the general population: a systematic review. J Affect Disord.

[4] Nguyen HC, Nguyen MH, Do BN, Tran CQ, Nguyen TTP, Pham KM (2020). People with suspected COVID-19 symptoms were more likely depressed and had lower health-related quality of life: the potential benefit of health literacy. J Clin Med.

[5] Rogers JP, Chesney E, Oliver D, Pollak TA, McGuire P, Fusar-Poli P (2020). Psychiatric and neuropsychiatric presentations associated with severe coronavirus infections: a systematic review and meta-analysis with comparison to the COVID-19 pandemic. Lancet Psychiatry.

[6] Rajkumar RP (2020). COVID-19 and mental health: a review of the existing literature. Asian J Psychiatr.

[7] Parola A, Rossi A, Tessitore F, Troisi G, Mannarini S (2020). Mental health through the COVID-19 quarantine: a growth curve analysis on Italian young adults. Front Psychol.

[8] Guerrini Usubini A, Cattivelli R, Varallo G, Castelnuovo G, Molinari E, Giusti EM (2021). The relationship between psychological distress during the second wave lockdown of COVID-19 and emotional eating in Italian young adults: the mediating role of emotional dysregulation. J Pers Med.

[9] Loffler-Stastka H, Bednar K, Pleschberger I, Prevendar T, Pietrabissa G (2021). How to include patients’ perspectives in the study of the mind: a review of studies on depression. Front Psychol.

[10] Cao W, Fang Z, Hou G, Han M, Xu X, Dong J (2020). The psychological impact of the COVID-19 epidemic on college students in China. Psychiatry Res.

[11] Pietrabissa G, Simpson SG (2020). Psychological consequences of social isolation during COVID-19 outbreak. Front Psychol.

[12] Giusti EM, Pedroli E, D'Aniello GE, Stramba Badiale C, Pietrabissa G, Manna C (2020). The psychological impact of the COVID-19 outbreak on health professionals: a cross-sectional study. Front Psychol.

[13] Ministero della Salute (2021). Covid-19 - Situazione nel mondo.

[14] Bertuzzi V, Semonella M, Bruno D, Manna C, Edbrook-Childs J, Giusti EM (2021). Psychological support interventions for healthcare providers and informal caregivers during the COVID-19 pandemic: a systematic review of the literature. Int J Environ Res Public Health.

[15] Rossi Ferrario S, Panzeri A, Cerutti P, Sacco D (2021). The psychological experience and intervention in post-acute COVID-19 inpatients. Neuropsychiatr Dis Treat.

[16] Pietrabissa G, Manzoni GM, Algeri D, Mazzucchelli L, Carella A, Pagnini F (2015). Facebook use as access facilitator for consulting psychology. Austr Psychol.

[17] Rossi AA, Marconi M, Taccini F, Verusio C, Mannarini S (2021). From fear to hopelessness: the buffering effect of patient-centered communication in a sample of oncological patients during COVID-19. Behav Sci (Basel).

[18] Panzeri A, Rossi Ferrario S, Cerutti P (2021). Psychological differences among healthcare workers of a rehabilitation institute during the COVID-19 pandemic: a two-step study. Front Psychol.

[19] Maunder RG (2009). Was SARS a mental health catastrophe?. Gen Hosp Psychiatry.

[20] Schwartz R, Sinskey JL, Anand U, Margolis RD (2020). Addressing postpandemic clinician mental health : a narrative review and conceptual framework. Ann Intern Med.

[21] Panzeri A, Rossi Ferrario S (2020). Supporting rehabilitation patients with COVID-19 during the pandemic: experiences from a technology-based psychological approach.

[22] Murphy R, Calugi S, Cooper Z, Dalle GR (2020). Challenges and opportunities for enhanced cognitive behaviour therapy (CBT-E) in light of COVID-19. Cogn Behav Therap.

[23] Jassi A, Shahriyarmolki K, Taylor T, Peile L, Challacombe F, Clark B (2020). OCD and COVID-19: a new frontier. Cogn Behav Therap.

[24] Murray H, Grey N, Wild J, Warnock-Parkes E, Kerr A, Clark DM (2020). Cognitive therapy for post-traumatic stress disorder following critical illness and intensive care unit admission. Cogn Behav Therap.

[25] Wang C, Pan R, Wan X, Tan Y, Xu L, Ho CS (2020). Immediate psychological responses and associated factors during the initial stage of the 2019 coronavirus disease (COVID-19) epidemic among the general population in China. Int J Environ Res Public Health.

[26] Kang L, Li Y, Hu S, Chen M, Yang C, Yang BX (2020). The mental health of medical workers in Wuhan, China dealing with the 2019 novel coronavirus. Lancet Psychiatry.

[27] Shangguan F, Quan X, Qian W, Zhou C, Zhang C, Zhang XY (2020). Prevalence and correlates of somatization in anxious individuals in a Chinese online crisis intervention during COVID-19 epidemic. J Affect Disord.

[28] Brog NA, Hegy JK, Berger T, Znoj H (2021). An internet-based self-help intervention for people with psychological distress due to COVID-19: study protocol for a randomized controlled trial. Trials..

[29] Wei N, Huang BC, Lu SJ, Hu JB, Zhou XY, Hu CC (2020). Efficacy of internet-based integrated intervention on depression and anxiety symptoms in patients with COVID-19. J Zhejiang Univ Sci B.

[30] Pizzoli SMF, Marzorati C, Mazzoni D, Pravettoni G (2020). An Internet-based intervention to alleviate stress during social isolation with guided relaxation and meditation: protocol for a randomized controlled trial. JMIR Res Protoc.

[31] Barak A, Hen L, Boniel-Nissim M, Shapira N (2008). A comprehensive review and a meta-analysis of the effectiveness of internet-based psychotherapeutic interventions. J Technol Hum Serv.

[32] Griffiths KM, Farrer L, Christensen H (2010). The efficacy of internet interventions for depression and anxiety disorders: a review of randomised controlled trials. Med J Austr.

[33] Andersson G (2018). Internet interventions: past, present and future. Internet Interv.

[34] Liu S, Yang L, Zhang C, Xiang YT, Liu Z, Hu S (2020). Online mental health services in China during the COVID-19 outbreak. Lancet Psychiatry.

[35] Castelnuovo G, Pietrabissa G, Manzoni GM, Sicurello F, Zoppis I, Molinari E (2020). Fighting the COVID-19 pandemic using the technology-based second-line in Italy and Lombardy: the urgent need of home-based remote monitoring systems to avoid the collapse of the hospital-centred first line. J Glob Health.

[36] Simpson S, Richardson L, Pietrabissa G, Castelnuovo G, Reid C (2021). Videotherapy and therapeutic alliance in the age of COVID-19. Clin Psychol Psychother.

[37] Shafran R, Clark DM, Fairburn CG, Arntz A, Barlow DH, Ehlers A (2009). Mind the gap: improving the dissemination of CBT. Behav Res Ther.

[38] Nobili RM, Gambazza S, Spada MS, Tutino AL, Bulfamante AM, Mariani A (2021). Remote support by multidisciplinary teams: a crucial means to cope with the psychological impact of the SARS-COV-2 pandemic on patients with cystic fibrosis and inflammatory bowel disease in Lombardia. Int J Clin Pract.

[39] Wang H, Zhao Q, Mu W, Rodriguez M, Qian M, Berger T (2020). The effect of shame on patients with social anxiety disorder in internet-based cognitive behavioral therapy: randomized controlled trial. JMIR Ment Health.

[40] Nordgreen T, Havik OE, Ost LG, Furmark T, Carlbring P, Andersson G (2012). Outcome predictors in guided and unguided self-help for social anxiety disorder. Behav Res Ther.

[41] Andersson G, Paxling B, Roch-Norlund P, Ostman G, Norgren A, Almlov J (2012). Internet-based psychodynamic versus cognitive behavioral guided self-help for generalized anxiety disorder: a randomized controlled trial. Psychother Psychosom.

[42] Van't Hof E, Cuijpers P, Stein DJ (2009). Self-help and Internet-guided interventions in depression and anxiety disorders: a systematic review of meta-analyses. CNS Spectr.

[43] Ciuca AM, Berger T, Crisan LG, Miclea M (2018). Internet-based treatment for panic disorder: a three-arm randomized controlled trial comparing guided (via real-time video sessions) with unguided self-help treatment and a waitlist control. PAXPD study results. J Anxiety Disord.

[44] Holst A, Nejati S, Bjorkelund C, Eriksson MC, Hange D, Kivi M (2017). Patients’ experiences of a computerised self-help program for treating depression - a qualitative study of Internet mediated cognitive behavioural therapy in primary care. Scand J Prim Health Care.

[45] Reins JA, Boss L, Lehr D, Berking M, Ebert DD (2019). The more I got, the less I need? Efficacy of Internet-based guided self-help compared to online psychoeducation for major depressive disorder. J Affect Disord.

[46] Vernmark K, Lenndin J, Bjarehed J, Carlsson M, Karlsson J, Oberg J (2010). Internet administered guided self-help versus individualized e-mail therapy: a randomized trial of two versions of CBT for major depression. Behav Res Ther.

[47] Andersson E, Enander J, Andren P, Hedman E, Ljotsson B, Hursti T (2012). Internet-based cognitive behaviour therapy for obsessive-compulsive disorder: a randomized controlled trial. Psychol Med.

[48] Ye YY, Chen NK, Chen J, Liu J, Lin L, Liu YZ (2016). Internet-based cognitive-behavioural therapy for insomnia (ICBT-i): a meta-analysis of randomised controlled trials. BMJ Open.

[49] Coull G, Morris PG (2011). The clinical effectiveness of CBT-based guided self-help interventions for anxiety and depressive disorders: a systematic review. Psychol Med.

[50] Balestrieri M, Carta MG, Leonetti S, Sebastiani G, Starace F, Bellantuono C (2004). Recognition of depression and appropriateness of antidepressant treatment in Italian primary care. Soc Psychiatry Psychiatr Epidemiol.

[51] Goldberg DP (1972). The detection of psychiatric illness by questionnaire.

[52] American Psychiatric Association. Diagnostic and statistical manual of mental disorders. 5th ed. Washington, DC: Routledge; 2013.

[53] Faul F, Erdfelder E, Buchner A, Lang AG (2009). Statistical power analyses using G*Power 3.1: tests for correlation and regression analyses. Behav Res Methods.

[54] Pietrabissa G, Castelnuovo G, Jackson JB, Rossi A, Manzoni GM, Gibson P (2019). Brief strategic therapy for bulimia nervosa and binge eating disorder: a clinical and research protocol. Front Psychol.

[55] Cattivelli R, Castelnuovo G, Musetti A, Varallo G, Spatola CAM, Riboni FV (2018). ACTonHEALTH study protocol: promoting psychological flexibility with activity tracker and mHealth tools to foster healthful lifestyle for obesity and other chronic health conditions. Trials..

[56] Cohen J. In: Press A, editor. Statistical power analysis for the behavioral sciences. London; 2013.

[57] Eid M, Gollwitzer M, Schmitt M (2017). Statistik und forschungsmethoden.

[58] Castelnuovo G, Manzoni GM, Villa V, Cesa GL, Pietrabissa G, Molinari E (2011). The STRATOB study: design of a randomized controlled clinical trial of Cognitive Behavioral Therapy and Brief Strategic Therapy with telecare in patients with obesity and binge-eating disorder referred to residential nutritional rehabilitation. Trials..

[59] Chiappelli M, Lo Coco G, Gullo S, Bensi L, Prestano C (2008). The Outcome Questionnaire 45.2. Italian validation of an instrument for the assessment of psychological treatments. Epidemiol Psichiatr Soc.

[60] Mondo M, Sechi C, Cabras C. Psychometric evaluation of three versions of the Italian Perceived Stress Scale. Curr Psychol. 2019;40(4):1–9.

[61] Balzarotti S, John OP, Gross JJ (2010). An Italian Adaptation of the Emotion Regulation Questionnaire. Eur J Psychol Assess.

[62] Bottesi G, Ghisi M, Altoè G, Conforti E, Melli G, Sica C (2015). The Italian version of the Depression Anxiety Stress Scales-21: Factor structure and psychometric properties on community and clinical samples. Compr Psychiatry.

[63] Wang C, Tee M, Roy AE, Fardin MA, Srichokchatchawan W, Habib HA (2021). The impact of COVID-19 pandemic on physical and mental health of Asians: a study of seven middle-income countries in Asia. PLoS One.

[64] Wang C, Tripp C, Sears SF, Xu L, Tan Y, Zhou D (2021). The impact of the COVID-19 pandemic on physical and mental health in the two largest economies in the world: a comparison between the United States and China. J Behav Med.

[65] Wang C, Lopez-Nunez MI, Pan R, Wan X, Tan Y, Xu L (2021). The impact of the COVID-19 pandemic on physical and mental health in China and Spain: cross-sectional study. JMIR Form Res.

[66] Soraci P, Ferrari A, Abbiati FA, Del Fante E, De Pace R, Urso A, et al. Validation and psychometric evaluation of the Italian version of the fear of COVID-19 scale. Int J Ment Heal Addict. 2020;20(4):1–10. 10.1007/s11469-020-00277-1 PMC7198091 32372892

[67] Sibilia L, Schwarzer R, Jerusalem M (1995). Italian Adaptation of the General Self-Efficacy Scale: Self-Efficacy Generalizzata.

[68] De Girolamo G, Rucci P, Scocco P, Becchi A, Coppa F, D'Addario A (2000). Quality of life assessment: validation of the Italian version of the WHOQOL-Brief. Epidemiol Psichiatr Soc.

[69] Brooke J, Jordan PW, Thomas B, Weerdmeester BA, McClelland IL (1996). SUS: A “quick and dirty” usability scale. Usability evaluation in industry.

[70] Vlaescu G, Alasjo A, Miloff A, Carlbring P, Andersson G (2016). Features and functionality of the Iterapi platform for internet-based psychological treatment. Internet Interv.

[71] Semonella M, Vilchinsky N, Dakel R, Biliunaite I, Pietrabissa G, Andersson G (2020). SOSteniamoci: an internet-based intervention to support informal caregivers. PSYCHOBIT.

[72] Thoren ES, Oberg M, Wanstrom G, Andersson G, Lunner T (2014). A randomized controlled trial evaluating the effects of online rehabilitative intervention for adult hearing-aid users. Int J Audiol.

[73] Hesser H, Gustafsson T, Lunden C, Henrikson O, Fattahi K, Johnsson E (2012). A randomized controlled trial of Internet-delivered cognitive behavior therapy and acceptance and commitment therapy in the treatment of tinnitus. J Consult Clin Psychol.

[74] Tulbure BT, Szentagotai A, David O, Stefan S, Mansson KN, David D (2015). Internet-delivered cognitive-behavioral therapy for social anxiety disorder in Romania: a randomized controlled trial. PLoS One.

[75] Johansson R, Sjoberg E, Sjogren M, Johnsson E, Carlbring P, Andersson T (2012). Tailored vs. standardized internet-based cognitive behavior therapy for depression and comorbid symptoms: a randomized controlled trial. PLoS One.

[76] NIC A, IBM Corp (2016). IBM SPSS Statistics for Windows, Version 24.0.

[77] Barak A, Grohol JM (2011). Current and future trends in internet-supported mental health interventions. J Technol Hum Serv.

[78] Newman MG, Szkodny LE, Llera SJ, Przeworski A (2011). A review of technology-assisted self-help and minimal contact therapies for anxiety and depression: is human contact necessary for therapeutic efficacy?. Clin Psychol Rev.

[79] Liverpool S, Mota CP, Sales CMD, Cus A, Carletto S, Hancheva C (2020). Engaging children and young people in digital mental health interventions: systematic review of modes of delivery, facilitators, and barriers. J Med Internet Res.

[80] Botella C, Mira A, Herrero R, García-Palacios A, Baños R (2015). Un programa de intervención auto-aplicado a través de Internet para el tratamiento de la depresión: “Sonreír es divertido”. Aloma..

[81] Castelnuovo G, Pietrabissa G, Cattivelli R, Manzoni GM, Molinari E (2016). Not only clinical efficacy in psychological treatments: clinical psychology must promote cost-benefit, cost-effectiveness, and cost-utility analysis. Front Psychol.

