# Correction: Study protocol for a randomized controlled trial evaluating the effectiveness of an internet-based self-help intervention to cope with psychological distress due to COVID-19 in the Italian general population: the RinasciMENTE project

**DOI:** 10.1186/s13063-023-07217-z

**Published:** 2023-03-17

**Authors:** Vanessa Bertuzzi, Michelle Semonella, Gerhard Andersson, Gian Mauro Manzoni, Gianluca Castelnuovo, Enrico Molinari, Giada Pietrabissa

**Affiliations:** 1 Department of Psychology, Catholic University of Milan, 20123 Milan, Italy; 2 Department of Psychology, Bar-Ilan University, 52900 Ramat-Gan, Israel; 3 Department of Behavioural Science and Learning, Linköping, Sweden; 4 Department of Clinical Neuroscience, Karolinska Institute, Solna, Sweden; 5 Department of Psychology, Faculty of Psychology, eCampus University, 22100 Como, Italy; 6 Psychology Research Laboratory, Istituto Auxologico Italiano IRCCS, Milan, Italy

**Correction: Trials 23, 801 (2022)**

**https://doi.org/10.1186/s13063-022-06714-x**

Following publication of the original article [1], the authors advised that affiliation 7 (now removed) was a duplicate of affiliation 1. The affiliation information has now been corrected in the published article. The authors thank you for reading this erratum and apologize for any inconvenience caused.
